# How erastin assassinates cells by ferroptosis revealed

**DOI:** 10.1093/procel/pwac007

**Published:** 2022-10-14

**Authors:** Boyi Gan

**Affiliations:** Department of Experimental Radiation Oncology, The University of Texas MD Anderson Cancer Center, Houston, TX 77030, USA

Ferroptosis represents an oxidative form of iron-dependent cell death resulting from unrestrained peroxidized phospholipids on cellular membranes (Jiang et al., 2021). In recent years, research interest in ferroptosis has increased steeply partly due to its high relevance to diverse diseases (such as cancer and neurodegenerative diseases) and the enormous potential of ferroptosis inducers and inhibitors to treat these diseases (Jiang et al., 2021; Tang et al., 2021; Lei et al., 2022). Remarkably, the research on ferroptosis originated from years of exploration on a small molecule compound called erastin (Dolma et al., 2003). In 2012, Dixon et al. reported that erastin can induce a form of iron-dependent cell death by inhibiting system X_c_^−^-mediated cystine import; they further coined a term ferroptosis to highlight the iron-dependent nature of this cell death (We now know that this is because chemical reactions in lipid peroxidation involve the Fenton reaction) (Dixon et al., 2012).

**Figure 1.**
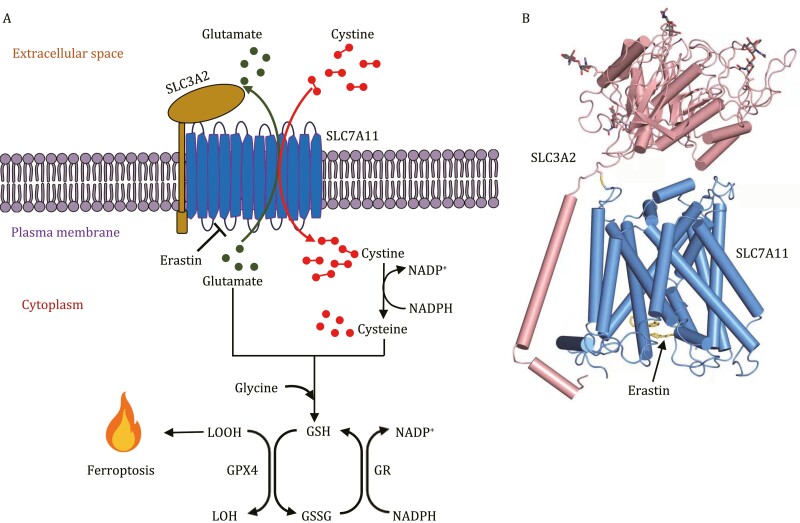
Erastin inhibits system X_c_^−^-mediated cystine transport and induces ferroptosis. (A) System X_c_^−^ consists of SLC7A11 and SLC3A2. SLC7A11 imports cystine and exports glutamate. Intracellular cystine is reduced to cysteine for GSH synthesis. GPX4 utilizes GSH as its co-factor to reduce LOOH to LOH and suppress ferroptosis, during which GSH is oxidized to GSSG, and GSSG is subsequently converted back to GSH by GR at the expense of NADPH. By blocking SLC7A11-mediated cystine import, erastin depletes intracellular GSH pools, leading to excessive lipid peroxidation and ferroptosis. (B) The atomic model of the erastin-bound SLC7A11-SLC3A2 complex. GPX4, glutathione peroxidase 4; GR, glutathione reductase; GSH, reduced glutathione; GSSG, oxidized glutathione; LOOH, lipid hydroperoxide; LOH, lipid alcohol; SLC3A2, solute carrier family 3 member 2; SLC7A11, solute carrier family 7 member 11.

System X_c_^−^ is an antiporter (a type of amino acid transporter that imports an amino acid and exports another amino acid) that composes of two subunits, including the transporter subunit SLC7A11 (also known as xCT) and the regulatory subunit SLC3A2 (also known as 4F2hc) (Fig. 1A) (Koppula et al., 2018). SLC7A11 imports cystine (an oxidized form of two cysteine molecules linked by a disulfide bond) into cells and exports glutamate out of cells at a 1꞉1 ratio, whereas SLC3A2 helps maintain the protein stability and subcellular localization of SLC7A11 (Fig. 1A) (Koppula et al., 2021). Once imported into the cytosol, cystine is reduced to cysteine for subsequent synthesis of glutathione (GSH), which is then used as a key cofactor by glutathione peroxidase 4 (GPX4) to detoxify lipid peroxides accumulated on cellular membranes, thereby mitigating ferroptosis (Fig. 1A) (Yang et al., 2014). The system X_c_^−^-GSH-GPX4 signaling axis constitutes the major defense mechanism against ferroptosis in mammalian cells. When erastin blocks the transporter activity of system X_c_^−^, the collapse of ferroptosis defense systems leads to the excessive accumulation of lipid peroxides on cell membranes and subsequent ferroptotic cell death (Jiang et al., 2021).

Erastin is the most widely used ferroptosis inducer in research, and erastin and its analogs (such as imidazole ketone erastin [IKE]) have been explored as anticancer drugs by triggering ferroptosis in cancer cells, although erastin has poor metabolic stability and solubility *in vivo*, limiting its ability to inducing ferroptosis in tumors (Feng and Stockwell 2018). The design of erastin analogs with improved potency and metabolic stability *in vivo* would improve its potential application as an anticancer drug but requires a deeper molecular understanding of erastin-mediated inhibitory effects on system X_c_^−^; however, the structural basis of how erastin inhibits system X_c_^−^ has remained a mystery. On the tenth anniversary of the discovery of ferroptosis, this mystery was recently solved by Yan et al. through cryo-electron microscopy (cryo-EM) analyses of the structure of the erastin-bound SLC7A11–SLC3A2 complex (Yan et al., 2022).

Cryo-EM analyses showed that, in the SLC7A11–SLC3A2 complex, SLC7A11 is a 12-pass transmembrane protein with an inward-facing conformation, whereas SLC3A2 is a single transmembrane protein and interacts with SLC7A11 at both transmembrane and extracellular regions through hydrophobic and polar interactions as well as disulfide bonding between these two proteins (Fig. 1B) (Yan et al., 2022). Further structural analyses revealed erastin binding to SLC7A11, wherein erastin is sandwiched between several transmembrane regions in SLC7A11 (Fig. 1B). This allowed the authors to identify the precise amino acid residues in SLC7A11 that mediate its binding to erastin, including F254 residue in the transmembrane 6b domain of SLC7A11 that interacts with the chlorophenoxy group in erastin (Yan et al., 2022).

Mutation of such amino acids in SLC7A11 would be expected to disrupt its interaction with erastin and therefore to make cells resistant to erastin-induced ferroptosis. Consistent with this, restoration of SLC7A11 F254A mutant in SLC7A11-deficient cells rendered cells much more resistant to erastin-induced lipid peroxidation and ferroptosis than did SLC7A11-deficient cells restored with wild-type SLC7A11 (Yan et al., 2022). Molecular docking analyses showed that IKE, an erastin analogue, can be docked in the same pocket in SLC7A11 as erastin; likewise, cells expressing SLC7A11 F254A mutant were more resistant to IKE-induced ferroptosis than were wild-type counterparts. Interestingly, F254A mutation in SLC7A11 did not affect ferroptosis induced by cystine starvation or sulfasalazine (another SLC7A11 inhibitor) (Yan et al., 2022), suggesting that this mutation does not affect SLC7A11’s ability to import cystine or the inhibitory effect of sulfasalazine on SLC7A11.

It should be noted that, while F254A mutation in SLC7A11 promotes cellular resistance to erastin, SLC7A11 F254A mutant-expressing cells still succumb to ferroptosis with erastin treatment at higher doses (Yan et al., 2022). It therefore appears that F254A mutation weakens but does not completely abolish SLC7A11 binding to erastin. Unfortunately, due to technical reasons, the current study failed to convincingly demonstrate the interaction between erastin and SLC7A11. Cells with the expression of SLC7A11 F254A mutant indeed exhibited more resistance to erastin-induced inhibitory effect on cystine uptake than did wild-type counterparts; however, eastin at higher doses still potently suppressed cystine uptake in F254A mutant-expressing cells (Yan et al., 2022), suggesting that F254A mutation only partially affects erastin’s ability to block SLC7A11.

Overall, this study will have a far-reaching impact on ferroptosis research at both fundamental and translational levels. First, the structure information will allow researchers to design various versions of SLC7A11 mutants (which, e.g., lack the ability to import cystine or do not interact with erastin or SLC3A2) for future functional investigations of ferroptosis. Since F254A mutation only moderately impairs the ability of erastin to block SLC7A11-mediated cystine transport, it is likely that mutation of multiple amino acids in SLC7A11 is required to fully abolish erastin binding to SLC7A11. Future studies should be directed to identifying such mutants of SLC7A11. In addition, exactly how erastin binding to SLC7A11 suppresses SLC7A11’s ability to import cystine (such as by inducing a conformational change in SLC7A11) is unclear from this study and remains an interesting topic for future investigations. Finally, IKE is considered a more potent ferroptosis inducer than erastin (Feng and Stockwell 2018). Interestingly, molecular docking analyses revealed that, compared to erastin binding to SLC7A11, IKE occupies a new sub-pocket in SLC7A11, indicating a stronger binding of IKE to SLC7A11 than erastin binding to SLC7A11, which might partly explains the potency of IKE in inducing ferroptosis. Therefore, the structure of the erastin-bound SLC7A11 will inspire further research to design better versions of erastin as ferroptosis inducers and anti-cancer drugs.
